# Dose-escalating ruxolitinib for refractory hemophagocytic lymphohistiocytosis

**DOI:** 10.3389/fimmu.2023.1211655

**Published:** 2023-06-29

**Authors:** Yue Song, Xiaoli Li, Xuefeng He, Fei Zhou, Feng Du, Ziyan Wang, Suning Chen, Depei Wu

**Affiliations:** ^1^ National Clinical Research Center for Hematologic Diseases, Jiangsu Institute of Hematology, The First Affiliated Hospital of Soochow University, Suzhou, China; ^2^ Institute of Blood and Marrow Transplantation, Collaborative Innovation Center of Hematology, Soochow University, Suzhou, China; ^3^ Department of Hematology, Soochow Hopes Hematonosis Hospital, Suzhou, Jiangsu, China

**Keywords:** ruxolitinib, JAK1/2 inhibitor, cytokines, hemophagocytic lymphohistiocytosis, dose-escalating, salvage therapy, stratification

## Abstract

**Background:**

Hemophagocytic lymphohistiocytosis (HLH) is a severe disorder characterized by excessive secretion of cytokines. Even with the recommended HLH-94/2004 regimen, over 30% of patients remain refractory to frontline therapy or relapse after an initial response, leading to poor clinical outcomes. Ruxolitinib, a JAK1/2 inhibitor targets key cytokines in HLH, has shown promising therapeutic effects. However, there has been little attention given to patients who do not respond to ruxolitinib and whether an escalating dose can provide a resolution.

**Methods:**

This study analyzed eight HLH patients who received dose-escalating ruxolitinib who had previously failed to respond to the general dose. The efficacy and safety were mainly analyzed.

**Results:**

Overall, four out of eight (50%) patients achieved better remission after dose escalation. Two patients who only showed improvement with the general dose achieved complete remission (CR) after dose escalation, and the other two patients also achieved CR after dose escalation when they did not respond to the general dose. The median time to achieve the best overall response was 18.5 days (IQR 13.25–23.75 days). There was no correlation of treatment outcome with blood count, liver function, LDH, cytokines, ferritin levels, NK cell activity, or the time to initiation of ruxolitinib and maximum dosage. The etiology of HLH (p=0.029) and level of sCD25 (p=0.021) correlated with treatment response to dose-escalating ruxolitinib. The area of sCD25 under the ROC curve was 0.8125 (95% CI 0.5921 to 1.033, p=0.035) when using 10,000 pg/ml as the cut-off value for predicting therapeutic effects. After a median follow-up of 159 days, two patients died, and the estimated 2-month overall survival rate was 75%. Adverse effects possibly related to the dose-escalating of ruxolitinib included two cases of extremity pain and one of aminotransferase increased. No grade 3 or higher adverse events were reported.

**Conclusion:**

This is the first comprehensive study on the use of dose-escalating ruxolitinib in HLH. Ruxolitinib at an escalated dose represent a viable and relatively safe solution for managing refractory HLH. The levels of sCD25 (with a cut-off of 10000pg/ml) can serve as an indicator for early consideration of chemotherapy during treatment.

## Introduction

Hemophagocytic lymphohistiocytosis (HLH) is a severe or even fatal hyperinflammatory disorder caused by a hereditary or acquired immunoregulatory abnormality, non-malignant proliferation of lymphocytes and tissue cells, and secretion of numerous inflammatory cytokines. Even with the recommended HLH-94/2004 regimen (1, 2), over 30% of HLH cases remain refractory to frontline therapy or relapse after an initial response, leading to poor clinical outcomes. Additionally, the toxicity of chemotherapy cannot be tolerated well in all types of patients, particularly those with organ failure (3, 4). The final common pathway in HLH pathogenesis is characterized by the overproduction of T-cell-derived cytokines, including interferon-γ (IFN-γ), IL-6, and others, as well as the phosphorylation-dependent activation of the Janus family kinases JAK1 and JAK2 (5). Blocking the JAK-STAT pathway shows promise in more effectively and safely reducing HLH-associated immunopathology by dampening downstream signaling of numerous HLH-associated cytokines and reducing chemotherapeutic toxicity.

Numerous JAK inhibitors, such as ruxolitinib, tofacitinib, baricitinib, and oclacitinib have been utilized for treating inflammatory disorders. Both experimental studies and early clinical reports have indicated that ruxolitinib may have utility in experimental and clinical HLH. Since the first confirmation of activity of ruxolitinib in HLH mouse models by Das et al. and Maschalidi et al. in 2016, an increasing number of publications describing its use in HLH patients (6, 7). Overall, ruxolitinib is well-tolerated and demonstrates prominent efficacy in active HLH (5). However, there has been a lack of concentration on patients who do not respond to ruxolitinib treatment, and previous clinical studies have shown that most ruxolitinib treatments result in partial remission, rather than complete remission (8, 9). Inadequate depth of remission may be related to the insufficient dosing. Ruxolitinib is a definite dose-dependent drug, as the phosphorylation of JAK and STAT proteins is dose-dependent (10, 11). In current clinical studies, the dosage of ruxolitinib is generally 10-15mg, twice a day. There has been no research exploring the application of escalating the dosage of ruxolitinib in HLH. It is well established that whether HLH can achieve remission is significantly related to prognosis, and patients who respond better to treatment often have a better prognosis. Is it possible that increasing doses of ruxolitinib can provide a better response in refractory patients and improve the depth of remission with higher doses of ruxolitinib? Also, whether increasing the dose of ruxolitinib will bring about more adverse events and how well it is tolerated. Therefore, we conducted this study to explore the dose-dependence of the therapeutic effect and safety of ruxolitinib in HLH.

## Methods

### Patients

We conducted a retrospective analysis of clinical data from 8 patients with HLH who received treatment with ruxolitinib at our center. HLH was diagnosed based on the HLH-2004 diagnostic criteria (1). EBV-HLH was diagnosed using DNA polymerase chain reaction (PCR) testing with a threshold of 10^3^ EBV-DNA copies per milliliter in plasma for confirmation of EBV infection. Fluorodeoxyglucose–positron emission tomography/computed tomography and bone marrow smear were performed for diagnosis of malignancy-associated HLH. For the diagnosis of familial HLH, a panel of 26 HLH-related genes was analyzed through next-generation sequencing. All patients provided written informed consent to receive treatment with Ruxolitinib and for blood sample collection.

### Treatment and efficacy

The treatment regimen of the ruxolitinib dosage consisted of two sequential phases: general-dose treatment with ruxolitinib, followed by individualized intensive treatment with escalated-dose ruxolitinib. Initially, all patients received oral ruxolitinib at a starting dose of 10/15 mg twice daily. In certain cases, corticosteroid, intravenous immunoglobulin (IVIG), and/or continuous renal replacement therapy were administered with ruxolitinib, but no chemotherapy was given before or during ruxolitinib treatment. Disease response evaluations were conducted at day 3, 7, 14, and 28 after ruxolitinib treatment. For patients with unfavorable responses (such as no improvement after 3 days of treatment, disease improvement but not achieving a favorable response, or disease progression at any time during general-dose treatment), the ruxolitinib dosage was escalated as determined by the clinicians based on the patient’s condition. The rate of dose escalation and the maximum dosage were tailored to each patient’s needs.

The efficacy evaluation of the treatment was mainly based on the criteria previously described in studies for HLH (12, 13). A complete response (CR) required normalization of all signs and laboratory abnormalities associated with HLH. A partial response (PR) was defined as meeting the criteria for CR in three or more clinical and laboratory abnormalities associated with HLH. Improvement in measures of HLH was defined as a change of greater than 50% from baseline in at least three clinical and laboratory abnormalities associated with HLH if these values were not normalized. Failure to achieve at least HLH improvement was defined as no response (NR). The interval to achieve best overall response was defined as the interval from the start of the ruxolitinib therapy to the recorded best response.

### Safety

Ruxolitinib’s main adverse effects include the potential for leukopenia, thrombocytopenia, elevated transaminases, elevated bilirubin, elevated triglycerides, infection (especially in pneumonia and urinary tract) (5, 14). Related indicators were monitored to evaluate the safety of therapy. Adverse events were graded and attributed in accordance with the National Cancer Institute Guidelines for the Cancer Therapy Evaluation Program.

### Outcome

The primary assessment was the overall response rate (ORR) to ruxolitinib treatment, which included the proportion of patients achieving complete response (CR), partial response (PR), and improvement in HLH measures. The safety and tolerability of ruxolitinib treatment, survival at 2 months from the first dose of ruxolitinib, time to response, interval to achieve the best response, changes in pharmacodynamic biomarkers, such as inflammatory cytokines, between baseline and post-treatment, and the cause of death, were also collected and analyzed.

### Statistical analysis

Survival was calculated from the date of diagnosis until the date of death from any cause or the date of the last follow-up. GraphPad Prism 9.0 and SPSS 22.0 (IBM, New York/USA) statistical software was adopted, and data that did not fit a normal distribution are presented as median and range. T-test was used for data that fit a normal distribution and homogeneity of variance, and Wilcoxon rank sum test was used for others. The median follow-up time was estimated with the use of Kaplan-Meier methods. All tests were two-sided, and a P value of 0.05 was considered to indicate statistical significance.

## Results

### Patients and clinical manifestation

A total of 8 patients with HLH were included, with a median age of 46.5 years (range, 27-76 years), comprising of 2 male and 6 females. HLH was secondary to malignancy (n=4, 2 of myelodysplastic syndrome and 2 of lymphoma), autoimmune disorder (n=3), or pregnancy (n=1). Prior to receiving ruxolitinib, all patients presented with typical features of aggressive HLH, including persistent fever (8/8), cytopenia (8/8), hepatosplenomegaly (7/8), hypofibrinogenemia/hypertriglyceridemia (8/8), hemophagocytosis (5/8), low NK-cell activity (2/8), and elevated serum ferritin (8/8) and sCD25 (8/8) levels. Genetic testing showed heterozygous mutations in the PRF1 gene in 2 patients (patient 3 and 5) and LYST gene in 2 patients (patient 2 and 7).

Four of the enrolled patients had received prior treatment for HLH, including corticosteroids (2 patients), IVIG (1 patient), and a combination of corticosteroids, IVIG, and Cyclosporin A (1 patient). Of these patients, 2 did not respond to treatment, while the other 2 experienced transient improvements followed by rapid relapses. The median time from onset of HLH to initiation of ruxolitinib therapy was 18 days (range, 6-34 days). The clinical characteristics of all 8 patients are summarized in **Table 1**.

**Table 1:** Clinical characteristics of the patients.

Case	Gender	Age (years)	Clinical features	Aetiology	WBC (*10^9^/L)	PLT (*10^9^/L)	Fbg (g/L)	TG (mmol/L)	Ferritin (ng/ml)	sCD25 (pg/ml)	Hemophagocytosis	Low NK-cell activity	Genetic defects
1	F	29	Fever, splenomegaly, liver dysfunction	Autoimmune disorder	2.52	50	1.28	3.4	1298	3642	No	Yes	ND
2	F	32	Fever, splenomegaly, liver dysfunction	Adult-onset Still’s disease	1.27	174	2.57	2.09	4082	24737	Yes	No	LYST heterozygous
3	F	32	Fever, liver dysfunction	Systemic lupus erythematosus	1.57	35	0.73	2.99	2000	4249	No	Yes	PRF1 heterozygous
4	F	64	Fever, splenomegaly, liver dysfunction	Myelodysplastic syndrome	1.17	21	2.48	2.17	6537	85940	Yes	No	ND
5	F	27	Fever, splenomegaly, liver dysfunction	Pregnancy	2.45	25	2.64	2.04	17114	4399	Yes	No	PRF1 heterozygous
6	M	76	Fever, splenomegaly	Myelodysplastic syndrome	4.06	16	2.34	1.93	3139	26682	Yes	No	ND
7	F	61	Fever, splenomegaly, liver dysfunction	Lymphoma	8.55	27	1.85	3.57	1311	59803	Yes	No	LYST heterozygous
8	M	65	Fever, splenomegaly, liver dysfunction	Lymphoma	2.06	46	0.35	3.95	300000	40829	No	No	ND

F, female; M, male; ND, not detected; WBC, white blood cell; PLT, platelets count; Fbg, fibrinogen; TG, triglyceride; NK-cell, natural killer cell.

### Efficacy

Overall, 50% (4 out of 8) of the patients achieved better remission after receiving an escalated dose of ruxolitinib. Among them, patient 1 and 5 achieved HLH improvement at the general dose of ruxolitinib (10mg twice daily and 15mg twice daily, respectively), but complete remissions were obtained only after the dose of ruxolitinib was escalated to 20mg twice daily. Patients 2 and 3 did not respond to the general dose of ruxolitinib, but achieved complete remissions of HLH after the dose of ruxolitinib was increased to 20mg twice daily in combination with triazoles (CYP450 inhibitors to increase concentration of ruxolitinib (15)). The median time to achieve the best overall response was 18.5 days (IQR 13.25–23.75 days). In the remaining four patients, the general dose of ruxolitinib was ineffective, and the dose of ruxolitinib was increased to a maximum of 25mg twice daily (+ triazole) in patient 4, 30mg twice daily in patient 6, and 30mg three times a day in patients 7 and 8. Although two patients’ body temperature returned to normal after receiving an escalated dose of ruxolitinib, their laboratory indicators continued to deteriorate. A small dose of etoposide (<100mg/m^2^) was added in patient 7 and 8 after failure of escalated doses of ruxolitinib and both of them achieved remission.

**Figure 1:**
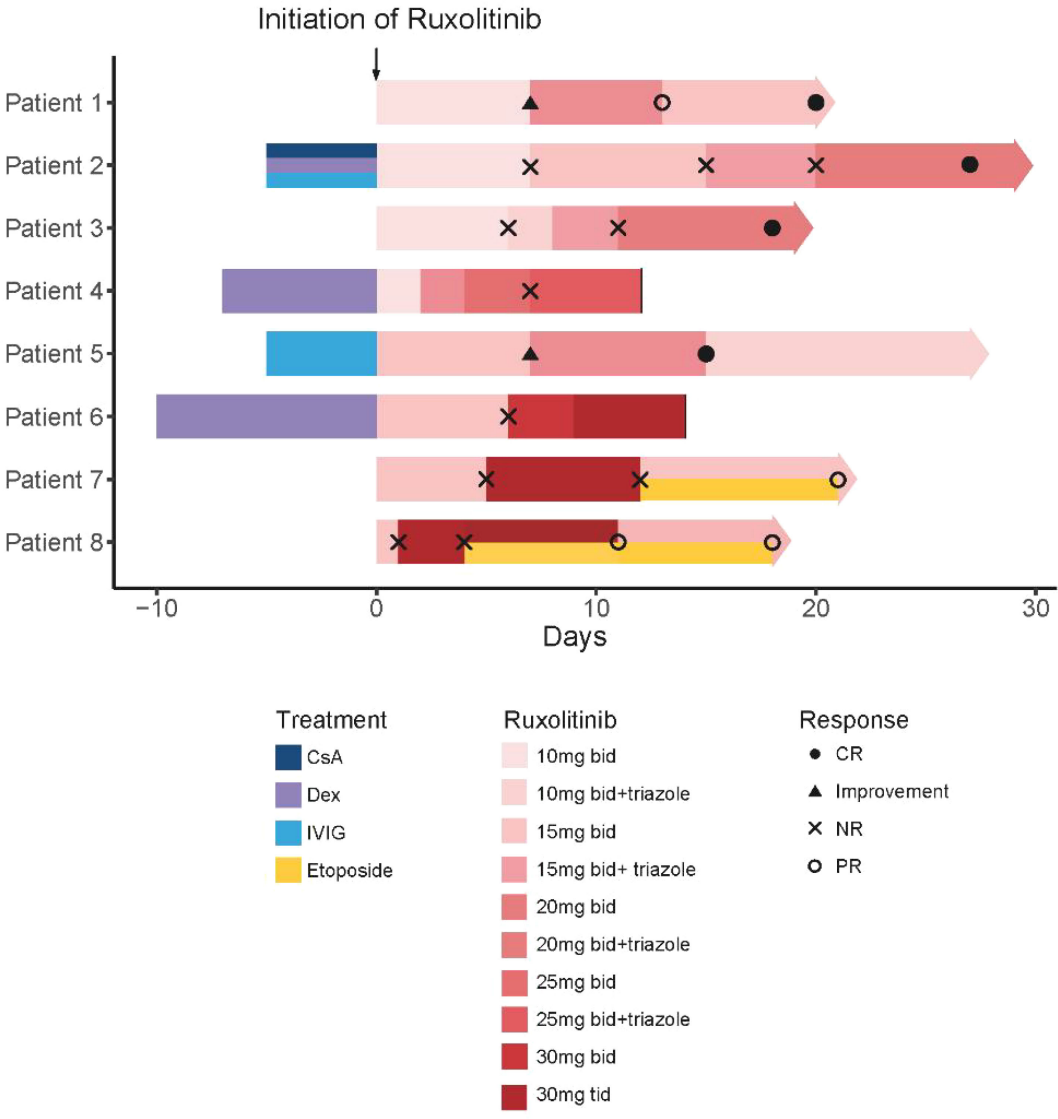
Swimmer plot of time on treatment of 8 HLH patients. Solid lines indicate that the patient died. CsA, cyclosporin A; Dex, dexamethasone; IVIG, intravenous immunoglobulin.

Regarding combination therapy, four patients received concomitant treatment with CRRT, two patients with malignancy-associated HLH were treated with BCL-2 inhibitors, and three patients received corticosteroids in addition to ruxolitinib. Three patients remained on corticosteroids upon initiation of ruxolitinib and effective treatment with ruxolitinib in one of them led to a reduction in corticosteroid use. Detailed information on treatment and efficacy can be found in **Table 2** and **Figure 1**.

**Table 2:** Treatment details and outcome of patients.

Case	Treatment before ruxolitinib	Onset of HLH to ruxolitinib	Initial dose of ruxolitinib	Response 1	Maximum dose of ruxolitinib	Response 2	Interval to achieve best response	Combined treatment	Outcome
1	N	6 days	10mg bid+triazoles	Improvement	20mg bid	CR	14 days	N	Survival
2	Coticosteroids+IVIG+CsA	22 days	10mg bid	NR	20mg bid+triazoles	CR	24 days	Corticosteroids	Survival
3	N	7 days	10mg bid	NR	20mg bid+triazoles	CR	23 days	Corticosteroids, CRRT	Survival
4	Corticosteroids	21 days	10mg bid	NR	25mg bid+triazoles	NR	–	Corticosteroids, CRRT, BCL-2 inhibitor	Death
5	IVIG	34 days	15mg bid	Improvement	20mg bid	CR	13 days	Corticosteroids, IVIG	Survival
6	Corticosteroids	22 days	15mg bid	NR	30mg bid	NR	–	Corticosteroids, BCL-2 inhibitor	Death
7	N	10 days	15mg bid	NR	30mg tid	Improvement	–	Corticosteroids, CRRT	Survival
8	N	15 days	15mg bid	NR	30mg tid	NR	–	Corticosteroids, CRRT	Survival

Triazoles, CYP450 inhibitors.

**Figure 2:**
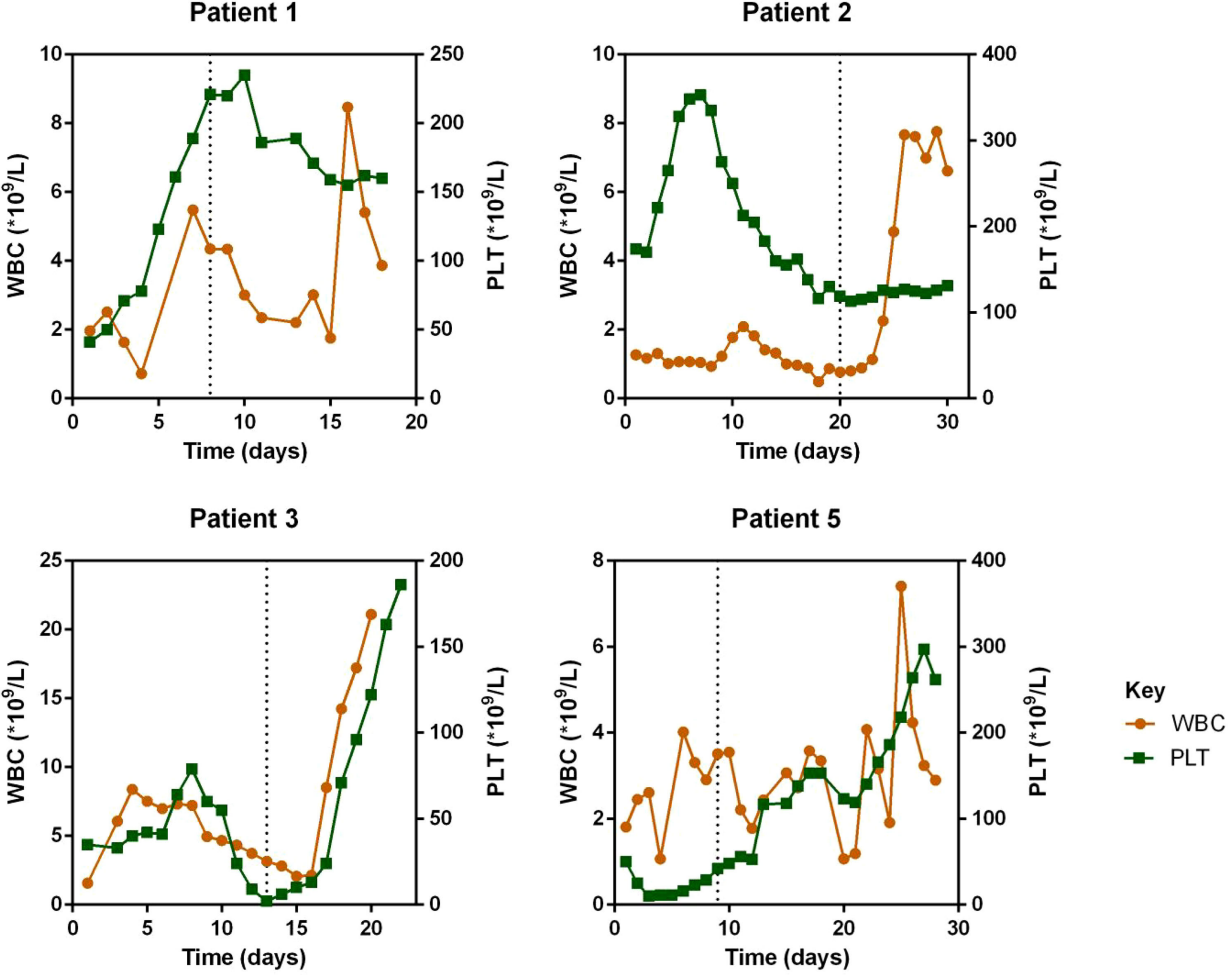
Counts of white blood cells (WBC) and platelets (PLT) before and after ruxolitinib in peripheral blood of responded patients. Initiation of ruxolitinib treatment occurred at day 0, the time point of maximum dose of escalating-ruxolitinib was indicated by the dotted line.

**Figure 3:**
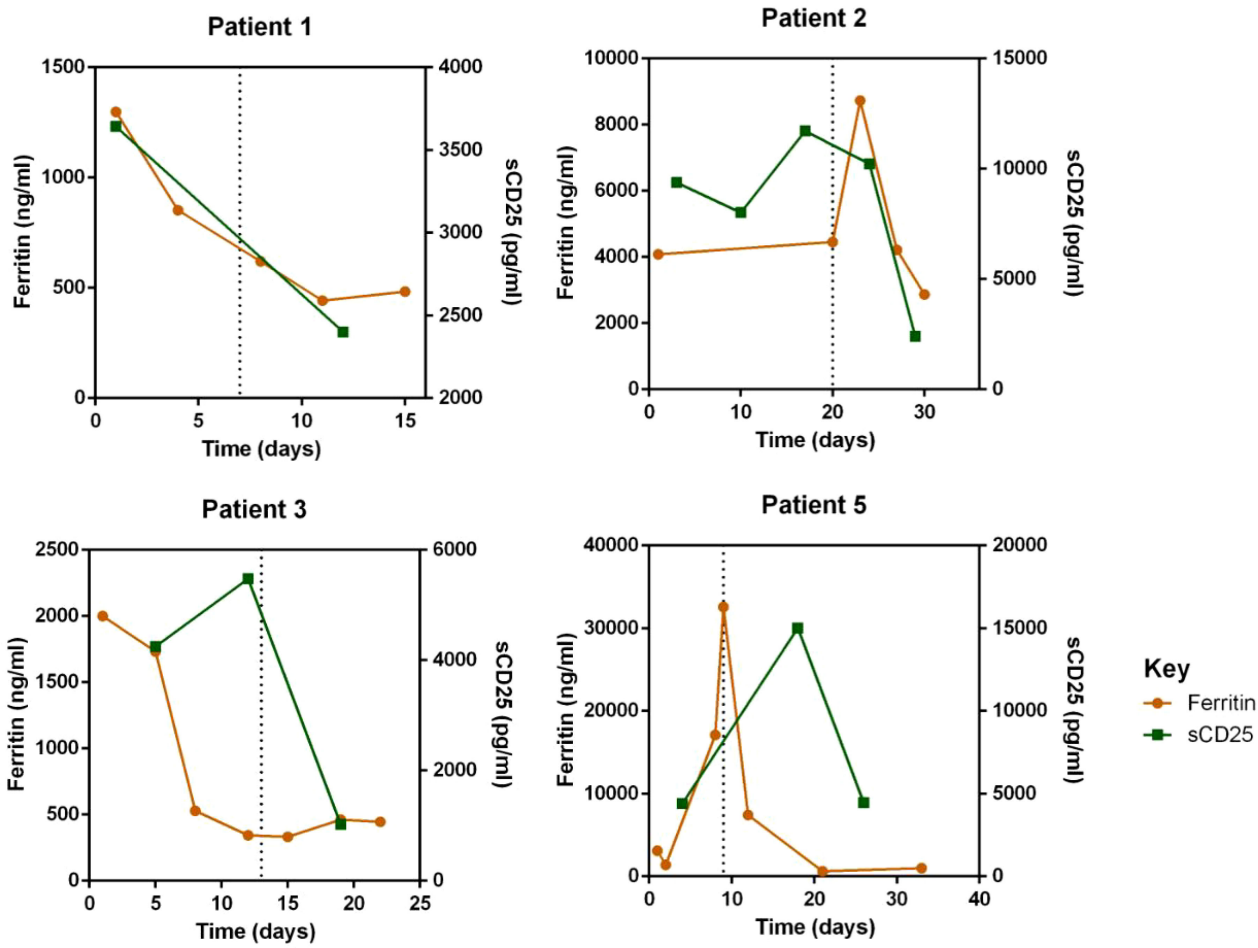
Levels of HLH markers: serum ferritin and soluble-CD25 (sCD25) before and after ruxolitinib in peripheral blood of responded patients. Initiation of ruxolitinib treatment occurred at day 0, the time point of maximum dose of escalating-ruxolitinib was indicated by the dotted line.

**Figure 4:**
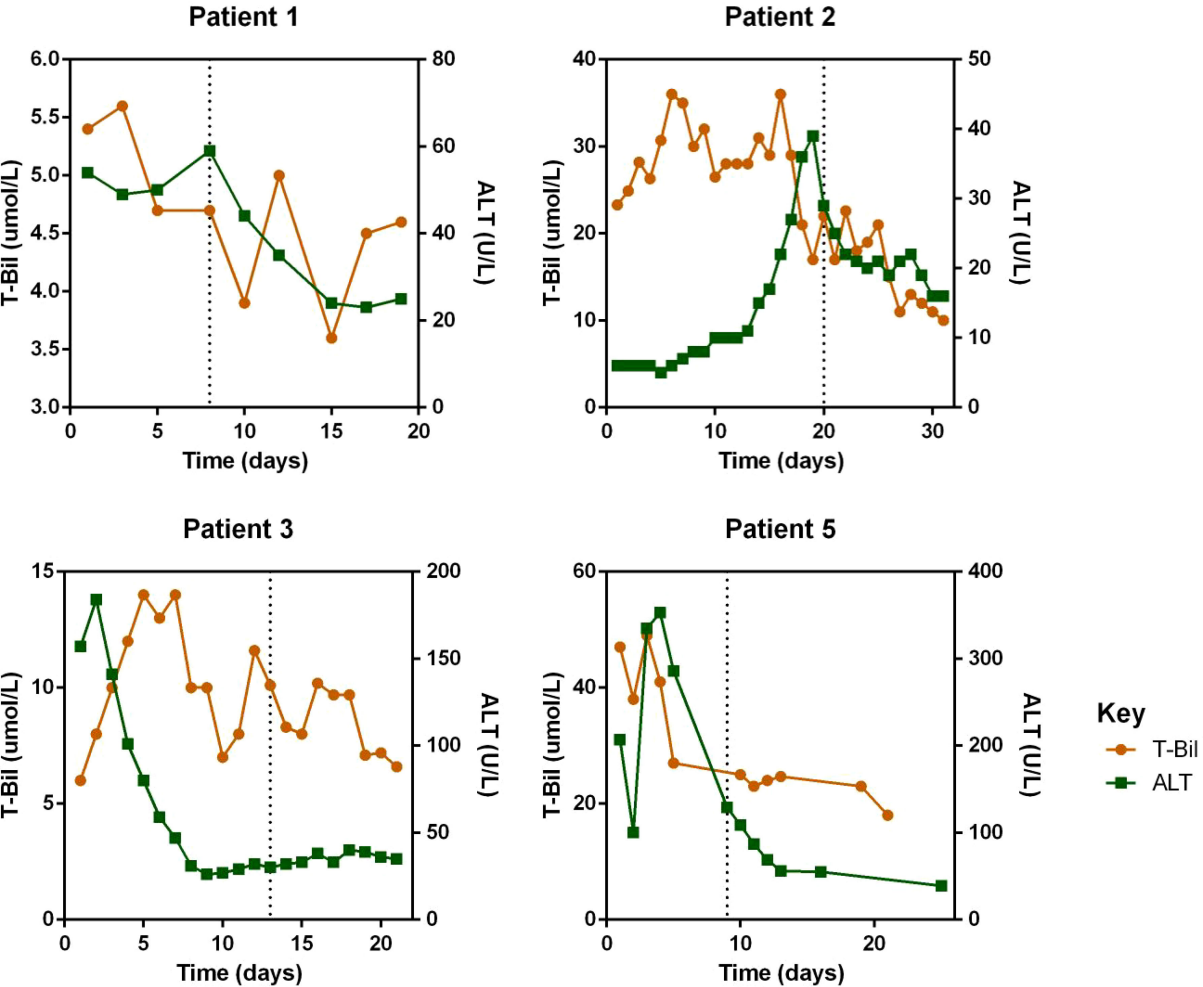
Levels of Alanine transaminase (ALT) and total bilirubin (T-Bil) before and after ruxolitinib in peripheral blood of responded patients. Initiation of ruxolitinib treatment occurred at day 0, the time point of maximum dose of escalating-ruxolitinib was indicated by the dotted line.

**Figure 5:**
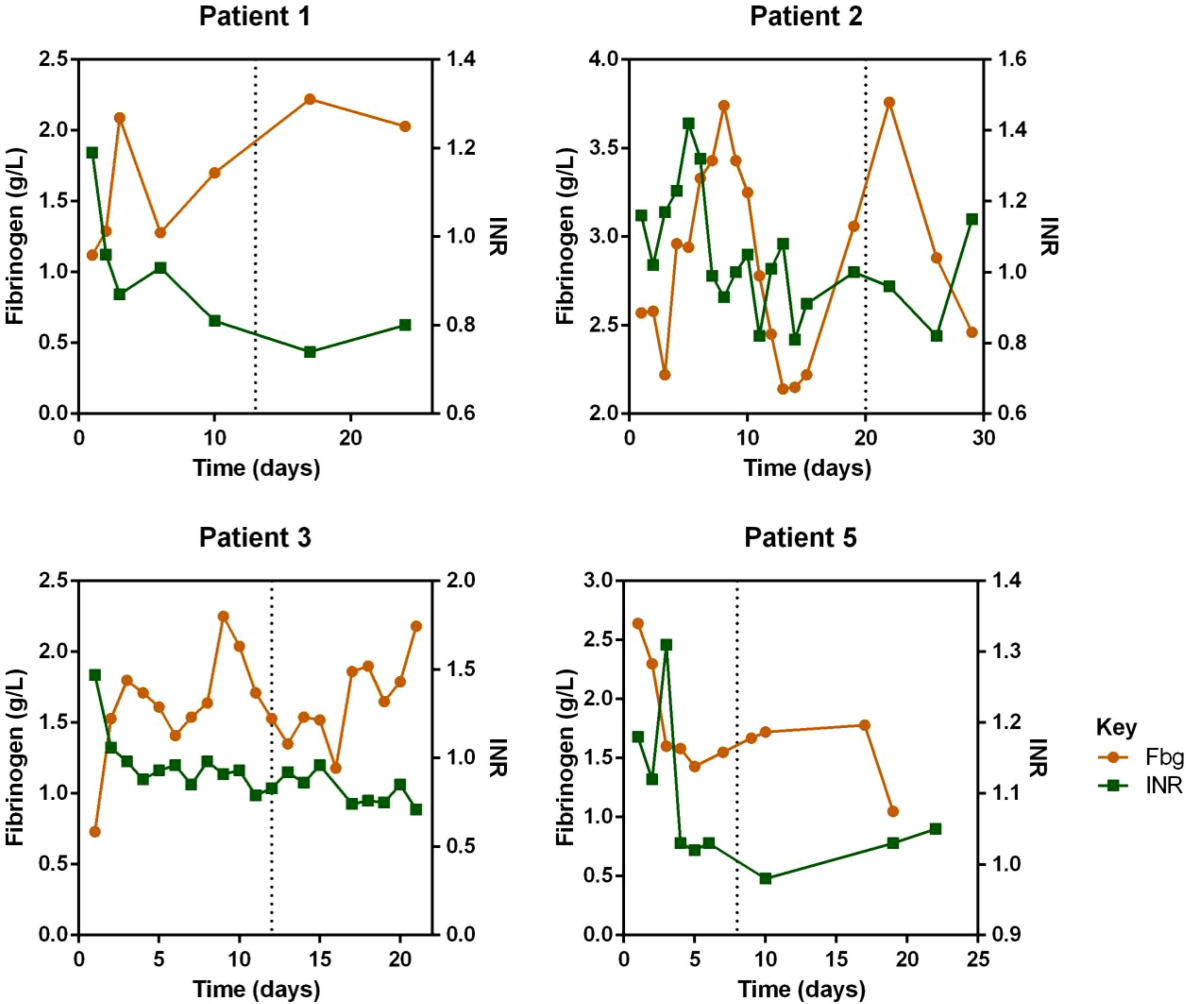
Levels of fibrinogen (Fbg) and international normalized ratio (INR) before and after ruxolitinib of responded patients. Initiation of ruxolitinib treatment occurred at day 0, the time point of maximum dose of escalating-ruxolitinib was indicated by the dotted line.

Regarding laboratory indicators, cytopenia was substantially resolved in all four patients who responded to escalated-dose ruxolitinib. In patient 1 and 5, the improvement in cytopenia after the general-dose of ruxolitinib was insufficient for remission. However, after escalating the dose of ruxolitinib, the counts of white blood cells and platelets recovered significantly. In patient 2 and 4, cytopenia did not improve after general-dose ruxolitinib treatment. But after escalating the dose of ruxolitinib, the counts of white blood cells and platelets increased significantly (as shown in **Figure 2**). Ferritin and sCD25 levels, HLH markers, substantially decreased in the four patients with effective escalated-dose ruxolitinib. It is noteworthy that in patient 1 and 5, both markers decreased further after the dose escalation, while in patients 2 and 3, the escalated dose of ruxolitinib led to decreases in ferritin and sCD25 levels that were not observed with general dosing (as shown in **Figure 3**). Patients with liver dysfunction experienced significant decreases in ALT and/or T-Bil levels after effective escalated-dose ruxolitinib treatment, but not in those who failed to respond (as shown in **Figure 4**). Patients’ coagulation function also improved with dose-escalating ruxolitinib and was presented in **Figure 5**.

### Outcome

**Figure 6:**
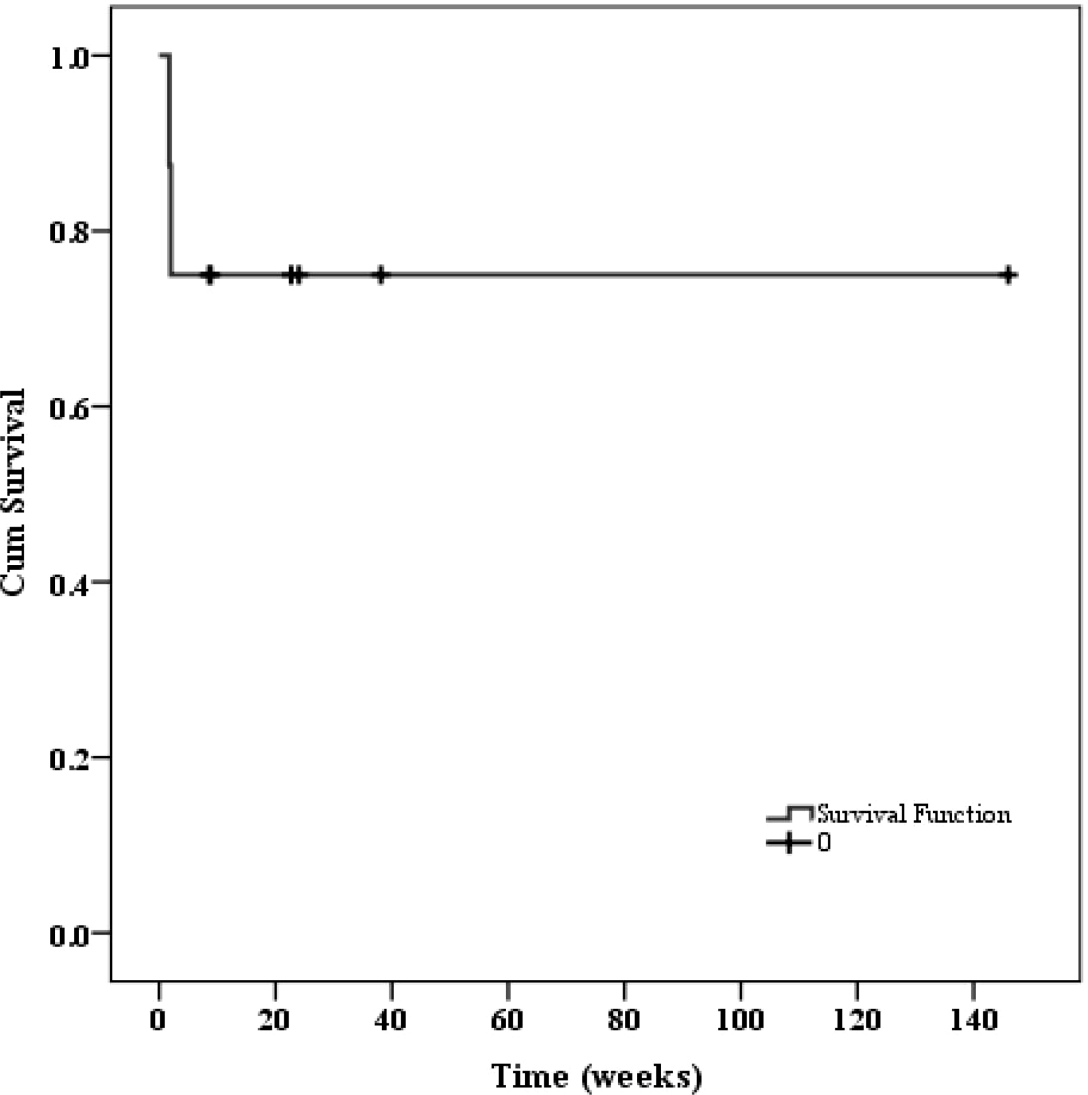
The survival function of all 8 patients.

Regarding patients who achieved remission, three received primary disease-specific treatment and one underwent pregnancy termination after remission of HLH. None of these four patients experienced HLH recurrence, and all survived until follow-up. Patient 7 and 8, who achieved remission after adding etoposide to high-dose ruxolitinib, also survived until follow-up. After a median follow-up of 159 days (IQR 25-242 days), two patients (patient 4 and 6) died, resulting in an estimated two-month overall survival rate of 75%. The survival curve was presented in **Figure 6**.

### Safety

No grade 3 or higher adverse events were observed in any of the patients during the study. Two patients experienced pulmonary infections while undergoing treatment, but they were not attributed to ruxolitinib since they occurred before the initiation of treatment. Two patients experienced aggravated aminotransferase increased, which may have been caused by the worsening of HLH in addition to drug-related factors. Two patients experienced grade 1 pain in the extremities, and one patient experienced skin rash.

Among these patients, adverse effects possibly related to the increased dose of ruxolitinib included two cases of extremity pain and one case of aminotransferase increased. However, no adverse events led to discontinuation of treatment and no other adverse effects were observed. Please refer to **Table 3** for further details.

**Table 3:** Adverse events possibly related to treatment.

Case	Type of adverse event reported	Grade	Attribution to ruxolitinib	Attribution to increased dosage	Event that led to discontinuation
1	Pain in extremity	1	Possible	Possible	No
2	Infection (pulmonary)	2	Un-related	Un-related	No
3	Infection (pulmonary)	2	Un-related	Un-related	No
	Skin rash	1	Possible	Un-related	No
4	ALT and AST increased	2	Possible	Possible	No
5	ND	–	–	–	–
6	ND	–	–	–	–
7	Pain in extremity	1	Possible	Possible	No
	ALT and AST increased	2	Possible	Un-related	No
8	ND	–	–	–	–

ALT, alanine aminotransferase; AST, aspartate aminotransferase.

### Comprehensive characterization of responders and non-responders

In order to investigate the factors that predict patient outcomes in HLH patients treated with dose-escalating ruxolitinib, we analyzed the correlation between response to treatment and common clinical and laboratory features before treatment. We found that most laboratory indicators, including blood cell counts, levels of AST, ALT, T-bil, TG, Fbg, LDH, ferritin, cytokines (IL-2, IL-4, IL-6, IL-10, IFN-5, TNF-α, IL-17A), NK cell activity or genetic defects, did not show any correlation with treatment outcome. In addition, the time from symptom onset to initiation of ruxolitinib treatment and the time to the use of the maximum dose of ruxolitinib were also not related to treatment effectiveness (as shown in **Table 4**).

**Table 4:** Comparison of patients’ characteristics between responders and non-responders before treatment.

Clinical features	Responders	Non-responders	p valve
Age, years			
Median	30.5	64.5	0.020
Range	[27, 32]	[61, 76]	
Gender			0.429
Male (n)	0	2	
Female (n)	4	2	
HLH etiology			0.029
Non-malignancy	4	0	
Malignancy	0	4	
WBC (*10^9^/L)	2.94 ± 2.14	3.96 ± 3.29	0.623
HGB (g/L)	82 ± 15	93 ± 27	0.516
PLT (*10^9^/L)	37 [25, 174]	24 [16, 46]	0.248
ALT (U/L)	119 [6, 335]	34.5 [21, 517]	0.773
AST (U/L)	177 [9, 1146]	89.5 [20, 1454]	0.773
Total bilirubin (µmol/L)	16.5 [5.4, 49.0]	14.8 [13.6, 359.2]	0.564
TG (mmol/L)	2.56 ± 0.76	2.91 ± 1.00	0.601
Fbg (g/L)	2.01 ± 0.70	1.76 ± 0.74	0.692
LDH (U/L)	1119.5 [243, 1951]	924 [279, 6410]	0.773
Ferritin (ng/mL)	3041 [1298, 17114]	4838 [1311, 300000]	0.564
sCD25 (pg/mL)	4324 [3642, 24737]	50316 [26682, 85940]	0.021
IL-2 (pg/ml)	0.10 [0, 2.52]	0 [0, 0.8]	0.508
IL-6 (pg/ml)	67.50 [9.00, 200.85]	52.30 [29.10, 428.3]	0.564
IL-10 (pg/ml)	10.11 [0, 43.70]	1514.65 [350.20, 5973.20]	0.130
TNF-α (pg/ml)	0.35 [0, 12.21]	0 [0, 0.60]	0.282
IFN-γ (pg/ml)	8.87 [0, 16.1]	40.30 [4.2, 114.4]	0.564
IL-17A (pg/ml)	3.95 [0, 36.99]	0 [0, 22.7]	0.508
Decreased NK cell activity (n)	0	2	0.429
Genetic defects related to HLH (n)	3	1	0.486
To Ru initiation (days)^*^	17.3 ± 13.4	15 ± 5.6	0.974
To Ru max-dose (days)^#^	30.3 ± 13.6	22.5 ± 8.4	0.371

the valve was expressed as median [range] or mean ± standard deviation.

^*^ To Ru initiation= Time from symptom onset to first ruxolitinib treatment.

^#^ To Ru max-dose= Time from symptom onset to escalation of the maximum dose of ruxolitinib.

**Figure 7:**
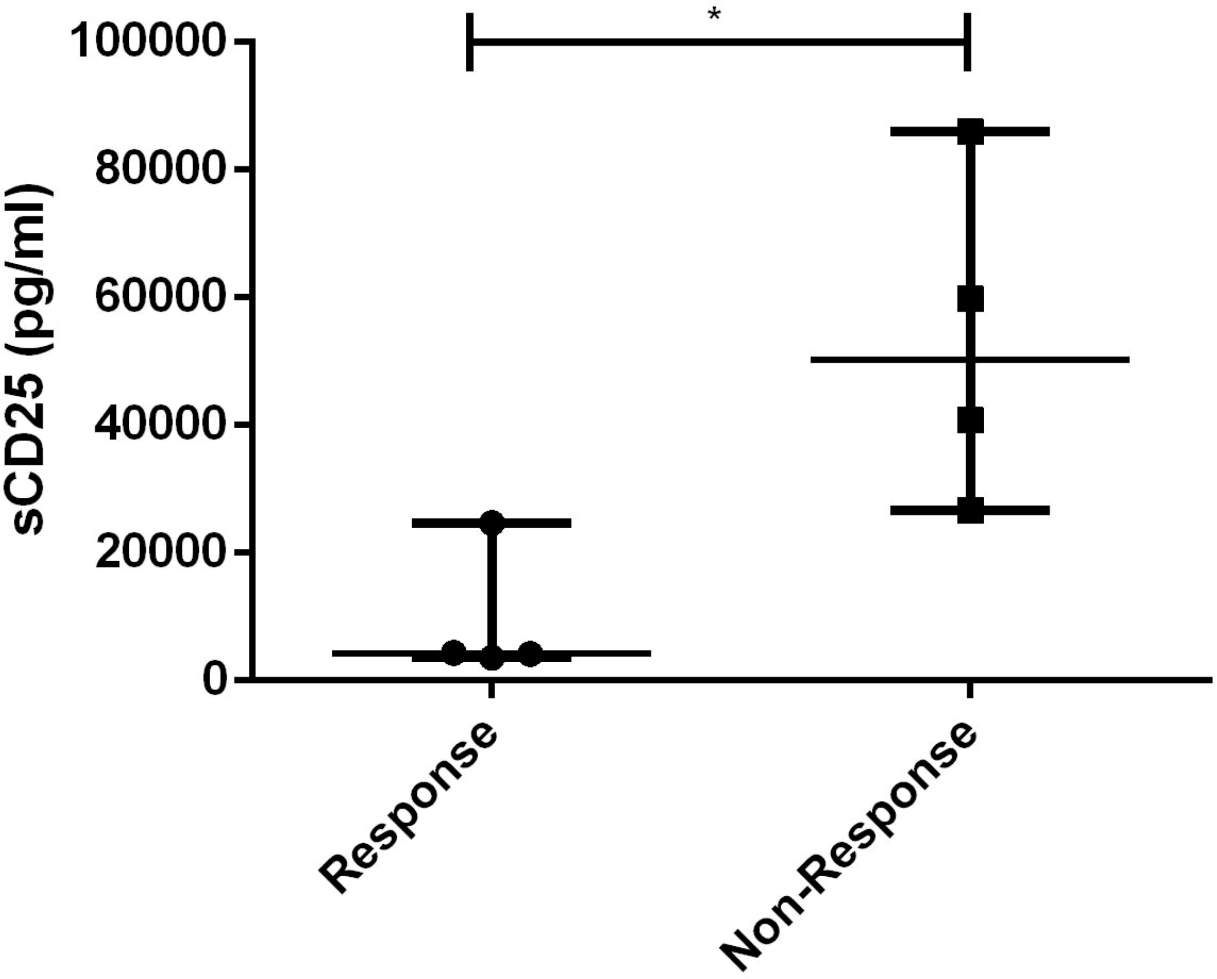
Levels of sCD25 before ruxolitinib between response and non-response to dose-escalating group. *, p<0.05.

The only laboratory indicator that showed a statistically significant difference between responders and non-responders to dose-escalating ruxolitinib was the level of sCD25 (p=0.021), as shown in **Figure 7**. Furthermore, using a cut-off value of 10000pg/ml, the area of sCD25 under the ROC curve was 0.8125 (95% CI 0.5921 to 1.033, p=0.035). Besides, HLH related to non-malignant diseases has a better response to escalating doses of ruxolitinib (p=0.029).

## Discussion

This study represents the first extensive investigation of the application of escalated doses of ruxolitinib for treating HLH. In this study, we observed that dose-escalating ruxolitinib demonstrated a significant impact in achieving improved remission among refractory HLH patients, with manageable adverse effects. Four of the eight patients achieved a sustained complete response with resolution of all features of the disease without relapse during the follow-up period. Remarkably, no serious drug-related severe adverse effects attributed to dose escalation were observed in any of the 8 patients. Two patients who failed to respond to dose-escalating ruxolitinib treatment also achieved remission after the addition of rather low-dose etoposide chemotherapy.

The therapeutic efficacy of ruxolitinib in HLH has been demonstrated in various ways (16, 17). However, the optimal dose and schedule of ruxolitinib administration remain to be determined. Despite individual case reports of using 20mg or even 25mg twice daily for HLH (18, 19), most formal clinical studies still use the conventional dose of 10-15mg twice daily (8, 9, 13, 20, 21). No studies have focused on the potential benefits of higher doses of ruxolitinib for HLH treatment. Nonetheless, studies focusing on other hematologic diseases have demonstrated the clinical benefits and tolerability of higher doses of ruxolitinib. In a clinical study by Zhang et al., patients were stratified for subsequent chemotherapy based on their response to front-line ruxolitinib (13). This study pointed out that due to the rapid deterioration of HLH, no ruxolitinib dose escalation set was performed, which might obscure the potential of high-dose ruxolitinib. Our study’s results confirmed that increasing the ruxolitinib dose had a positive effect on achieving remission or even deepening remission status for patients with an insufficient response. The mechanism underlying the efficacy of high-dose ruxolitinib in treating HLH may be attributed to its inhibitory effect on JAK kinases in a dose-dependent manner, given that ruxolitinib is a JAK1/2 inhibitor (10, 22). Several studies have demonstrated the dose-dependent nature of the inhibition of JAK kinases by JAK inhibitors in different diseases, such as Sjögren’s syndrome and rheumatoid arthritis, suggesting that higher doses can lead to better therapeutic outcomes (11, 23). In a recent study, it was discovered that ruxolitinib effectively ameliorates cytokines that signal through the JAK/STAT pathway, such as IL-6, IL-12, and IFN-γ, in experimental mouse models of HLH in a dose-dependent manner, with 60mg/kg showing significantly better results than 30mg/kg (24). These studys’ results provide a theoretical basis for the effectiveness of dose-escalating ruxolitinib in more effectively blocking the JAK-STAT pathway and better controlling HLH activity, which is confirmed by our clinical findings.

The heterogeneity of clinical efficacy of escalating doses of ruxolitinib needs to be noted. Obviously, not all refractory HLH patients respond well to dose-escalated ruxolitinib. Initiating jakinib therapy late in the disease process may not be sufficient to induce disease remission or to prevent life-threatening immune dysregulation (22). The disease burden in these patients was so great that even higher dose ruxolitinib is no longer effective. However, in our results, no correlation was seen between time of treatment initiation and treatment efficacy. The observed significant difference in the etiology of HLH between the responder and non-responder groups suggested that the etiology of HLH may be the underlying reason for the different therapeutic effects of escalating-doses of ruxolitinib. All of the four patients with poor response were diagnosed as malignancy associated HLH (M-HLH). It has been reported previously that patients with M-HLH did not carry a satisfactory respond to ruxolitinib. Patients with M-HLH may suffer from more severe cytokine storms due to the presence of malignancies, and the malignancy itself as an antigen may activate T cells through non-JAK-STAT related pathways, leading to downstream cytokine storms (25–27).

Interestingly, we observed that patients who did not respond to escalating-dose of ruxolitinib suffered significant higher sCD25 levels before treatment. When using 10,000pg/ml as the cutoff value, sCD25 can serve as an effective predictor for the therapeutic outcome of escalated-dose ruxolitinib. More notably, the sCD25 levels of patient 7 and 8 were significantly higher than 10,000pg/ml, and remission was successfully achieved by adding a relatively low dose of etoposide to the escalated-dose ruxolitinib treatment. These findings suggest that the levels of sCD25 may serve as an indicator for early consideration of chemotherapy during treatment: when sCD25 levels are below 10,000 pg/ml, escalating the dose of monotherapy with ruxolitinib can provide more effective remission of HLH; when sCD25 levels are above 10,000, chemotherapy drugs such as etoposide should be considered in addition to higher doses of ruxolitinib. Moreover, under conditions of high-dose ruxolitinib, chemotherapy drugs often require only a smaller dose to achieve the desired therapeutic effect. However, since sCD25 is known to be more elevated/predictive of diagnosis of malignancy, the observed difference in sCD25 levels may simply reflect the distinction between non-malignant and malignant conditions. To validate our conclusion, further investigation with a non-malignancy group is necessary. A prospective clinical trial (NCT04551131) is investigating the combination of ruxolitinib at a dose of 25 mg/m^2^ twice a day, along with dexamethasone and etoposide, in pediatric HLH patients. The trial is currently recruiting, and the results hold promise for future attention.

The increase in the dose of ruxolitinib, while improving the therapeutic effect, also brought concerns about safety. The main adverse effects of ruxolitinib are hematological toxicity, including neutropenia, thrombocytopenia and anemia. In the study of myelofibrosis, thrombocytopenia was identified as the dose-limiting toxic effect, and 25 mg twice daily and 100 mg once daily were identified as the maximum tolerated doses (14, 28). In other hematological diseases, the reported dose of ruxolitinib is up to 200mg twice daily, and the tolerance is still acceptable (29–31). In a phase II clinical trial using ruxolitinib in patients with relapsed/refractory leukemias, with a dose escalation to 50 mg twice daily, ruxolitinib was well tolerated. In this study, the maximum dose of ruxolitinib reached up to 30mg, three times a day, and no severe adverse effects related to dose-escalation was observed. Decreased blood counts and associated infections are probably the most worrisome side effects of high-dose ruxolitinib. It is noteworthy that most patients had significant cytopenia before the initiation of therapy and in none were these significantly worsened while on this study (32). In general, although recent studies have concerns that ruxolitinib’s inhibition of JAK2 will hamper hematopoiesis (12), according to the results of our study and previous reports, ruxolitinib will improve the cytopenia rather than worsen it, with HLH is under remarkable control.

## Conclusion

Our study suggests that escalating the dose of ruxolitinib can provide better resolution of refractory HLH with tolerable toxicity when the general dose fails. Upon a more comprehensive analysis to our data, it’s noted that ruxolitinib alone may not be enough for all patients and that addition of low dose chemotherapy (such as etoposide) may be helpful and is tolerated. The levels of sCD25 (with a cut-off of 10000pg/ml) may serve as an indicator for early consideration of chemotherapy during treatment of HLH. This is the first comprehensive study on the use of dose-escalating ruxolitinib in the treatment of HLH. Larger systematically protocol-driven prospective clinical trials and further mechanism studies are still necessary to validate our findings.

## Data availability statement

The raw data supporting the conclusions of this article will be made available by the authors, without undue reservation.

## Ethics statement

The studies involving human participants were reviewed and approved by Ethics Committee of The First Affiliated Hospital of Soochow University. The patients/participants provided their written informed consent to participate in this study.

## Author contributions

XH designed and supervised this study; SNC and DPW coordinated the study; YS and XL conducted the data analysis and wrote the manuscript. FZ, FD and ZW did research. All authors contributed to the article and approved the submitted version.
